# Using the Wilder Collaboration Factors Inventory to Strengthen Collaborations for Improving Maternal and Child Health

**DOI:** 10.1007/s10995-020-03091-2

**Published:** 2020-11-28

**Authors:** Rebecca Wells, Lindsey Yates, Isabel Morgan, Leslie deRosset, Dorothy Cilenti

**Affiliations:** 1 University of Texas School of Public Health, 1200 Pressler Street, Houston, 77030 USA; 2 Department of Health Policy and Management, University of North Carolina Gillings School of Global Public Health, McGavran-Greenberg Hall, CB# 7411, Chapel Hill, NC 27599-7411 USA; 3 Department of Maternal and Child Health, Gillings School of Global Public Health, University of North Carolina, McGavran-Greenberg Hall, CB# 7445, Chapel Hill, NC 27599-7411 USA; 4 Frank Porter Graham Child Development Center, The Impact Center, The University of North Carolina, 105 Smith Level Rd, CB 8180, Chapel Hill, NC 27599 USA; 5 Department of Maternal and Child Health, Gillings School of Global Public Health, University of North Carolina, 402A Rosenau Hall, CB#7445, Chapel Hill, NC 27599-7411 USA

**Keywords:** Leadership, Collaborative leadership, Measuring collaboration, Collective impact, Community capacity

## Abstract

**Introduction:**

The Wilder Collaboration Factors Inventory is a free, publicly available questionnaire about the quality and context of community collaboration. The purpose of this article is to share lessons from using this questionnaire in a North Carolina maternal and child health initiative.

**Methods:**

In 2015, the State’s General Assembly funded five local health departments to implement evidence-based strategies for improving maternal and child health. Each health department formed a community action team for this purpose. Members of each community action team completed the Wilder Collaboration Factors Inventory (Inventory) in the first year of funding and again 1 and 2 years later. Technical assistance coaches also asked community action team conveners to complete a brief questionnaire annually, and used these as well as Inventory results to plan for improvements.

**Results:**

During the first year, community action teams emerged as strong in seeing collaboration in their self-interest. A primary challenge noted by conveners was engaging consumers on the community action teams. Strategies to address this included using social media and compensating consumers for attending meetings. By the second year, teams’ average scores in engaging multiple layers of participation increased, and eight additional factors became strengths, which generally continued in year three. The most consistent challenge was supporting community action teams administratively.

**Discussion:**

The Wilder Collaboration Factors Inventory provided a feasible tool for identifying opportunities for improvement in several local, cross-sector partnerships, suggesting promise for other communities seeking to enhance their collective impact on maternal and child health.

## Significance

The systemic nature of factors affecting maternal and child health merits action by multiple sectors partnering with families. Decision makers are increasingly seeking to ensure active participation of all stakeholder groups in developing and implementing community strategies. The current study found the Wilder Collaboration Factors Inventory effective for helping coaches work with community action teams to improve their collaboration over time. The instrument was easy for participants to complete and yielded findings about specific collaboration factors that teams were able to use. Community leaders may find North Carolina’s experiences useful for comparison as they build and track their collaborative capacity.

## Introduction

Preterm birth and low birth weight are associated with long-term negative health conditions and high related spending for children (Behrman and Butler 2007; CDC 2018). Preterm birth and low birth weight rates have recently increased (Martin et al. 2018). There are also persistent disparities in these outcomes. For instance, the preterm birth rate is 50% higher for African American women than for white women (CDC 2018). Reasons for these increases are likely to have systemic origins, and hence require broad-based solutions (Black et al. 2017).

Funders increasingly require collaboration toward sustainable improvement (Gillam et al. 2016). One such example has been Improving Community Outcomes for Maternal and Child Health (ICO4MCH), funded by the North Carolina General Assembly (Morgan et al. 2020). ICO4MCH began with 2-year funding cycles, the first being June 1, 2016–May 31, 2018, from the state Division of Public Health to five local health departments with a combined service area of 14 counties. These funds were competitively awarded for implementing evidence-based strategies to lower infant mortality rates, improve birth outcomes, and improve the overall health of children ages birth to five.

The developers of ICO4MCH used a collective impact framework (Kania and Kramer 2011), implementation science (Fixsen et al. 2009, 2010), and a health equity lens. The funding required creation of a community action team in each site including health department staff, consumers, and other partners. These teams support implementation of evidence-based strategies and health equity to promote systems change, with a secondary goal of creating sustainable collaboration.

The intention of the collective impact framework is to overcome the usual “isolated impact” of actors with common goals who may not work together effectively, if at all. Instead, collective impact is envisioned as improving social outcomes at scale through intersectoral cooperation (Kania and Kramer 2011). In addition, the collective impact approach entails mutually reinforcing action by partners across sectors, as well as continuous learning. In the context of ICOMCH, this included collaboration within and beyond public health agencies. ICO4MCH also drew on implementation science, including an emphasis on ongoing feedback and coaching to participants in community innovation (Fixsen et al. 2005, 2009).

A central tool for providing ICO4MCH teams with feedback about their collaboration was the Wilder Collaboration Factors Inventory (Inventory). Technical Assistance (TA) coaches based in the University of North Carolina and State ICO4MCH leadership chose the Inventory as an easily interpretable instrument for tracking changes in perceived community action team collaboration over time. The Inventory draws on extensive reviews of prior research to assess factors previously found to be associated with success in collaboration, defined as a mutually beneficial relationship among organizations for common goals (Mattessich and Johnson 2016).

The purpose of the current paper is to describe lessons from the ICO4MCH program’s use of the Wilder Collaboration Factors Inventory that may apply to other communities implementing collective impact initiatives.

## Methods

### Procedure

TA coaches asked members of each community action team to complete the Wilder Collaboration Factors Inventory three times between 2016 and 2018: shortly after forming, and 12 and 24 months thereafter. Team members were given time to complete paper versions of the Inventory during meetings, as well as sent electronic links to the Inventory for completion at another time.

This project was conducted in accordance with prevailing ethical principles and reviewed by the University of North Carolina Institutional Review Board.

### Participants

Community action team members represent stakeholders invested in reducing adverse birth outcomes and improving child health. As specified by the authorizing legislation, these include individuals from women’s health and pediatric providers, local health department staff, and the supplemental nutrition program for women, infants, and children (WIC), as well as consumers living in the service areas who use public health and human services, and other community leaders. At least 25% of their members are consumers and represent local demographics.

### Assessment and Measures

The Wilder Collaboration Factors Inventory version used was that available in 2016, which entailed 40 items relating to 20 factors (Table 1). Those factors included participation by a range of stakeholders; clear roles and policies; sufficient resources for the participant time and administrative support collaboration requires; trust and commitment; and open and frequent communication (Ales et al. 2011; Gillam et al. 2016; Perrault et al. 2011). The study team made minor changes to Inventory item wording to enhance ease of reading without losing meaning. For instance, “collaborative” was sometimes replaced with “team” or omitted as an unnecessary qualifier, and “accomplish” was replaced with “do.” After this study was completed, a new version of the Inventory was released, with four more items reflecting two additional factors (evaluation and continuous learning and engaged stakeholders) (Wilder Foundation 2019).

**Table 1:** Wilder Collaborative Factors Inventory, 2016 Version

Factor	Items
History of collaboration or cooperation in community	1. Agencies in our community have history of working together 2. Trying to solve problems through collaboration has been common in this community. It’s been done a lot before
Collaborative group seen as a legitimate leader in the community	1. Leaders in this community who are not part of our **[‘collaborative’ deleted]** group seem hopeful about what we can **do** 2. Others (in this community) who are not a part of this **group** would generally agree that the organizations involved in this collaborative project are the “right” organizations to make this work
Favorable political and social climate	1. The political and social climate seems to be “right” for starting a collaborative project like this one 2. The time is right for this **[‘collaborative’ deleted]** project
Mutual respect, understanding and trust	1. People involved in our **group** always trust one another 2. I have a lot of respect for the other people involved in this **group**
Appropriate cross section of members	1. The people involved in our collaboration represent **all** those who have a stake in what we are trying to accomplish 2. All the organizations that we need to be members of this **[‘collaborative’ deleted]** group have become members of the group
Members see their collaboration as in their self-interest	1. My organization will benefit from being involved in this **project**
Ability to compromise	1. People involved in our **group** are willing to compromise on important aspects of our project
Members share a stake in both process and outcome	1. The organizations that belong to our **[‘collaborative’ deleted]** group invest the right amount of time in our collaborative efforts 2. Everyone who is a member of our **[‘collaborative’ deleted]** group wants this project to succeed 3. The level of commitment among the collaboration participants is high
Multiple layers of participation	1. When the collaborative group makes major decisions, there is always enough time for members to take information back to their organizations to **share** with colleagues about what the decision should be 2. Each of the people who participate in **[group deleted]** decisions in this collaborative group can speak for the entire organization they represent, not just a part
Flexibility	1. There is a lot of flexibility when decisions are made; people are open to discussing different options 2. People in this collaborative group are open to **talking about different options**
Development of clear roles and policy guidelines	1. People in this **[‘collaborative’ deleted]** group have a clear sense of their roles and responsibilities 2. There is a clear process for making decisions among the partners in this **group**
Adaptability	1. This **group** is able to adapt to **change**, such as fewer funds than expected, [‘changing political climate’ omitted] **leadership change** 2. This group **can** survive even if it had to make major changes in its plans or add some new members **[‘in order to’ deleted]** reach its goals
Appropriate pace of development	1. This **[‘collaborative’ deleted]** group has tried to take on the right amount of work at the right pace 2. We are currently able to **keep up with the work** necessary to coordinate all the people, organizations, and activities related to this collaborative project
Open and frequent communication	1. People in this **group** communicate openly with one another 2. I am informed as often as I should be about what goes on in the **group** 3. The people who lead this **[‘collaborative’ deleted]** group communicate well with the members
Established informal relationships and communication links	1. Communication among the people in this **[‘collaborative’ deleted]** group happens both at formal meetings and informal ways 2. I **[’personally’ deleted]** have informal **talks** about the project with others who are involved in this **[‘collaborative’ deleted]** group
Concrete, attainable goals and objectives	1. I **understand** what our **group** is trying to accomplish 2. People in our **[‘collaborative’ deleted]** group know and understand our goals 3. People in our **[‘collaborative’ deleted]** group have established reasonable goals
Shared vision	1. The people in this **[‘collaborative’ deleted]** group **believe** we can make this project work 2. My ideas about what we want to accomplish **[‘with this collaboration’ deleted]** seem to be the same as the ideas of others
Unique purpose	1. What we are trying to accomplish with our **[‘collaborative’ deleted]** project would be difficult for any single organization to accomplish **alone** 2. No other organization in the community is trying to do exactly what we are trying to do
Sufficient funds, staff, materials, and time	1. Our **[‘collaborative’ deleted]** group had adequate funds to do what it wants to **do** 2. Our **[‘collaborative’ deleted]** group has adequate “people power” to do what it wants to **do**
Skilled leadership	3. The **leaders of this effort** have good skills for working with other people and **groups**

Slight modifications for ease of reading are **bolded**

For each Inventory item, response options ranged from 1 (Strongly Disagree) to 5 (Strongly Agree). The score for each factor was calculated as the mean of all community action team members’ responses about the item(s) representing that factor. According to the Inventory developers, a score of 4.0 or above for a factor is a strength that does not require additional attention, factors with scores ranging from 3.0 to 3.9 are borderline and should be discussed by the group, and factors scoring 2.9 or below are areas of concern that should be addressed (Mattessich and Johnson 2016).

Each time after completing the Inventory, the TA coaches reflected with members of each community action team about how to interpret their scores, and how to build on strengths and identify opportunities for improvement. This entailed linking the team’s particularly high or low scores in the Inventory to the conditions for collective impact. For instance, coaches related team responses to Inventory items about concrete, attainable goals and objectives to the collective impact emphasis on a common agenda. These discussions included incorporating action steps into plans for the following year.

In addition, community action team conveners were asked open-ended questions each year: “What is working well in this group?” and “What needs improvement in this group?” Responses to these questions were content analyzed for insights about Inventory scores and related strategies.

## Results

Each of the five health departments represented a diverse area of North Carolina, with varying community characteristics, including the largest county in the state as well as smaller rural, urban, and mixed regions (US Census Bureau 2018) (Table 1). All teams sought to improve access to reproductive life planning, including long-acting reversible contraception. To reduce infant mortality, two teams implemented a tobacco reduction intervention; the other three supported breastfeeding. To improve children’s health, two teams addressed secondhand smoke exposure; two implemented parenting programs, and another implemented both a parenting program and nurse home visiting (Morgan et al. 2020).

TA coaches found the Inventory feasible to use, taking an average of 10 min for community action team members to complete. In the first year, 51 members across the five teams completed the Inventory, for an average of 10 respondents (ranging from 5 to 20) per site. In year 2, 60 team members completed the Inventory, for an average of 12 (ranging from 5 to 19) per site. In year 3, 78 team members completed the Inventory, for an average of 16 (ranging from 8 to 23) per site (Table 2).

**Table 2:** Characteristics of Sites in Improving Community Outcomes for Maternal and Child Health Initiative (ICO4MCH)

Community description	Urban single county	Rural multi-county	Urban multi-county	Rural multi-county	Urban–rural multi-county
Total Live Births^a^ (120,765 NC)	4231	1539	17,210	2214	7239
Infant mortality^b^ (7.1 NC)	5.9	7.8	6.0	9.0	9.8
Child poverty^c^, < 18 years. (23.4% NC)	26.0	26.9	17.8	43.5	29.3
Uninsured,^d^ < 19 years. (4.9 NC)	5.4	6.3	4.8	5.3	4.9
Preterm Births^e^ (%) (10.1 NC)	9.8	11.3	9.4	10.9	11.3
Low birthweight Rate^f^	9.0	9.2	9.1	12.6	11.1

Race/ethnicity,^g^					
White (%)	50.9	92.96	68.3	36.95	59.1
Black (%)	37.3	2.64	21.35	31.4	29.3
American Indian (%)	0.3	0.68	0.3	25.05	2.3
Asian (%)	4.8	0.5	3.95	0.7	1.6
Native Hawaiian/other Pacific Islander (%)	0	0.04	0.05	0	0.1
Hispanic (%)	13.5	5.84%	11.9	5.65	11.4
Household Median Income^h^	56,693	38,728	66,277	32,573	40,875

^a^NC Department of Health & Human Services State Center for Health Statistics, https://schs.dph.ncdhhs.gov/data/vital/volume1/2015/

^b^NC Department of Health & Human Services State Center for Health Statistics, https://schs.dph.ncdhhs.gov/data/mch/

^c^Children in Poverty in North Carolina, 2015, Kids Count Data Center, The Annie E. Casey Foundation, https://datacenter.kidscount.org/data/tables/2238-children-in-poverty#detailed/5/4910-5009/true/573/any/12873,4680

^d^Uninsured Population by Age Group, Children Under 19, 2017, Kids Count Data Center, The Annie E. Casey Foundation, https://datacenter.kidscount.org/data/tables/2281-uninsured-population-by-age-group?loc=35&loct=5#detailed/5/4910-5009/false/871,870,573,869,36,868,867,133,38,35/6192,6193,6194/4766,12956

^e^Percent of births less than 37 weeks (Preterm), Number and Percent of NC Resident Births Delivered by Gestation, 2013–2017, https://schs.dph.ncdhhs.gov/data/databook/CD7B%20Preterm%20births.html

^f^2015 North Carolina Vital Statistics, Volume 1, NC Department of Health and Human Services, https://schs.dph.ncdhhs.gov/data/vital/volume1/2015/

^g^Race, Hispanic or Latino and Race, U.S. Census Bureau, 2013–2017 American Community Survey 5-Year Estimates, https://factfinder.census.gov/faces/nav/jsf/pages/index.xhtml

^h^Economy, Median Household Income, ICO4MCH Social Determinants of Health Maps, https://unc.maps.arcgis.com/apps/MapSeries/index.html?appid=12c03017ad954d76a5d4c41d13ff26a9#

**Fig. 1:**
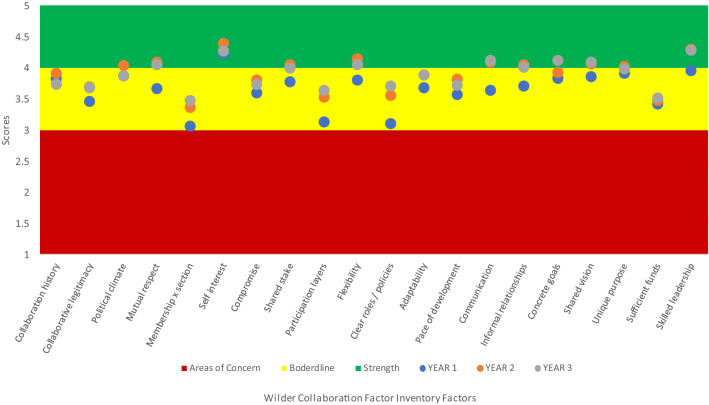
Wilder Collaboration Factors Inventory Average Scores across all sites each year

For each year we calculated the average scores for each factor across all sites (Table 3 and Fig. 1). In year 1, the only factor with an average score above 4.0 (‘strength’) was members seeing their collaboration as in their self-interest. Most factors had an average score between 3.0 and 3.9 (‘borderline’). The three factors with the lowest average scores were appropriate cross section of members, multiple layers of participation, and development of clear roles and policy guidelines. No factors averaged 2.9 or below (‘concern’).

**Table 3:**
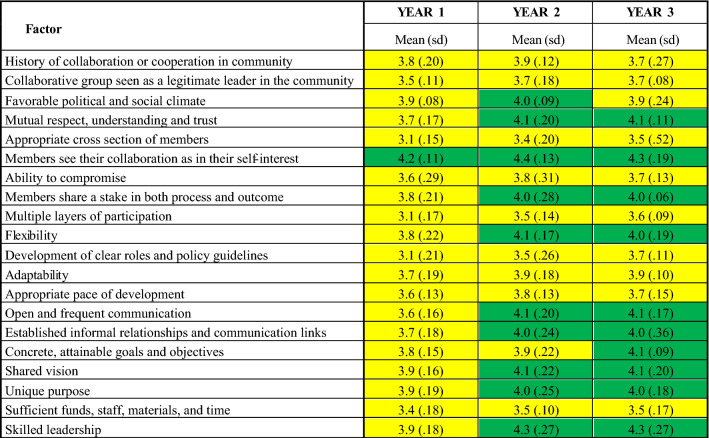
Wilder Collaboration Factors Inventory, Average Scores across all sites, Year 1–Year 3

Green: Scores 4.0 or above are strengths; do not need attention

Yellow: Scores 3.0–3.9 are borderline; should be discussed by the group

Inventory results were not used to choose interventions. Instead, Inventory results informed improvements in team functioning needed to achieve collective impact. The number of members in leadership roles was designated by the funder, and so did not change. However, the increasing numbers of people completing the Inventory over time suggest team growth.

In year 1, community action team convener responses to open-ended questions indicated perceptions of leaders and team members as passionate about their work. We interpreted these comments as related to the Inventory factor of members sharing a stake in processes and outcomes, which had a borderline average score in year 1 before becoming a strength in years 2 and 3.

However, members also reported that key stakeholders were missing from the teams, including providers as well as community members using services; we saw these comments as related to the Inventory factor about having an appropriate cross section of members, which tended to have borderline scores all 3 years. Further, members noted that their roles and specific team goals were unclear. These comments aligned with borderline average team scores all 3 years for the Inventory factor relating to development of clear roles and policy guidelines. Average scores for the related factor concerning concrete, attainable goals and objectives increased from borderline in years 1 and 2 to a strength in year 3.

[T]his project involves disparate health outcomes (e.g. tobacco cessation, family planning) and different members are involved in different parts of project so [it] is challenging to have [a] unified goal for collaborative; helpful to review/clarify what is needed from each partner, timeline of where we are going and of project goals (Year 1 single county urban site))

TA coaches helped members identify how to build on strengths and address areas for improvement. For instance, teams developed communication strategies including newsletters and using social media to increase visibility of ICO4MCH. Challenges in identifying clear roles and clarifying goals led teams to reevaluate their common agendas.

In year 2, members seeing collaboration as in their self-interest remained a strength across the five teams, and nine other factors also emerged as strengths, including mutual respect, understanding, and trust; flexibility; open and frequent communication; shared vision, and skilled leadership. In addition to the three lowest scoring factors from year 1, sufficient funds, staff, materials, and time also emerged as low scoring in year 2.

In year 3, 10 factors were again identified as strengths. Two factors shifted categories: favorable political and social climate moved from a strength to borderline; while concrete, attainable goals and objectives moved from borderline to a strength.

Community action team convener responses to open-ended questions in years 2 and 3 were consistent with year 1 responses. Common responses about what was working well included the energy and motivation of diverse participating members; improving coordination and communication; and good leadership. In addition, team conveners often noted location and meeting times, which is not in the Inventory.

The level of communication and how the leaders keep everyone in the group informed of our mission and the things that are being done from other organizations and programs. (Year 3 multicounty urban/rural site))

Common responses in years 2 and 3 about needed improvements included clear timelines and actions; identification of additional community members and agencies needed, including managers; and sustainable models of engagement.

Tangible goals and actions for group members to participate in both within and beyond the meeting setting. Additional consumers needed at the table. Co-leadership model is also in progress and would be ideal for sustainability purposes. (Year 2 multicounty rural site)).

In years 2 and 3 the TA coaches led the community action team members through additional strategic planning processes to address the lower scoring factors. The teams began implementing action steps over the year in an effort to address those factors. These included more use of social media to highlight team activities and targeted invitations to potential team members.

Overall, despite challenges identified by community action team members, by year 2, half of the factors across all sites were identified as strengths, which were largely sustained in year 3.

## Discussion

The experience reported here demonstrated the utility of the Wilder Collaboration Factors Inventory for improving maternal and child health community action teamwork over time. One of the lessons is the importance of editing the Inventory for ease of reading without losing the original meaning. It is also important to ensure that all members have access to the Inventory, evidenced in ICO4MCH through high and increasing survey participation rates over time. Study limitations include the absence of team members’ demographic information, which the Inventory does not address, and not having the number of team members in any given year.

Improving participant assessments of collaboration between years 1 and 2 suggest that tailored technical assistance using Inventory scores supported teamwork. In year 1, initial responses indicated shared perception that collaborating was in participants’ self-interest. By year 2, participants had slightly higher average assessments of such relational bases for successful collaboration as mutual respect and flexibility; evidence of improving collaboration, such as frequent and open communication (Gillam et al. 2016); and skilled leadership. In year 3 members continued to show enthusiasm for working together, valuing their comfort sharing information and mutual awareness achieved through meetings. ICO4MCH patterns fit those reported by other partnerships using the Inventory, such as initial exploratory participation, in search of increasing collective impact; early clarifications of participant roles and responsibilities paying off in later collaborative dynamics; and positive reinforcement over time between informal communication and trust (Ales et al. 2011; Gillam et al. 2016; Perrault et al. 2011).

Although four factors continued to have borderline average scores through year three, three of those four—appropriate cross section of members, multiple layers of participation, and development of clear roles and policies—did increase over time within that range. This progress is similar to that reported in prior research using the Inventory, such as initial identification of missing stakeholders and clarification of governance (Ales et al. 2011).

The one factor with an average score virtually unchanging from years one to three was sufficient funds, staff, materials, and time. Despite each of the partnerships receiving state funding to support the community action team and implement strategies, the continuing challenges relating to sustainability in ICO4MCH also mirror other community partnerships’ accounts of very substantial time and other resource requirements (Ales et al. 2011; Gillam et al. 2016). The collective impact framework encourages collaborative groups to have a backbone organization responsible for organizational and administrative support (Kania and Kramer 2011).

Finally, specifically aligning teams’ Wilder Collaboration Factors Inventory scores with collective impact goals can facilitate more effective collaboration. For instance, coaches noted how high Inventory scores in open and frequent communication were related to the continuous communication needed for collective impact (Kania and Kramer 2011). Similarly, coaches facilitated discussion of how the Inventory factor about awareness of mutual interest fit the collective impact framework’s emphasis on complementary activities. Results from this North Carolina initiative suggest that incorporating Inventory results into strategic action planning may help communities increase their collective impact in maternal and child health.
